# A retrospective study of myelin oligodendrocyte glycoprotein antibody-associated disease from a clinical laboratory perspective

**DOI:** 10.3389/fneur.2023.1187824

**Published:** 2023-09-12

**Authors:** Yufei Wang, Qusang Danzeng, Wencan Jiang, Bingqing Han, Xiaowen Zhu, Ziwei Liu, Jialu Sun, Kelin Chen, Guojun Zhang

**Affiliations:** ^1^Laboratory Diagnosis Center, Beijing Tiantan Hospital, Capital Medical University, Beijing, China; ^2^NMPA Key Laboratory for Quality Control of In Vitro Diagnostics, Beijing, China; ^3^Beijing Engineering Research Center of Immunological Reagents Clinical Research, Beijing, China

**Keywords:** MOGAD, multiple sclerosis, neuromyelitis optica spectrum disorders (NMOSD), laboratory data analysis, differential diagnosis

## Abstract

**Objectives:**

To analyze the differences in laboratory data between patients with myelin oligodendrocyte glycoprotein (MOG) antibody-associated disease (MOGAD), multiple sclerosis (MS) and neuromyelitis optica spectrum disorder (NMOSD).

**Methods:**

The study included 26 MOGAD patients who visited Beijing Tiantan Hospital from 2018 to 2021. MS and NMOSD patients who visited the clinic during the same period were selected as controls. Relevant indicators were compared between the MOGAD group and the MS/NMOSD groups, and the diagnostic performance of meaningful markers was assessed.

**Results:**

The MOGAD group showed a slight female preponderance of 57.7%, with an average onset age of 29.8 years. The absolute and relative counts of neutrophils were higher in the MOGAD group than in the MS group, while the proportion of lymphocytes was lower. The cerebrospinal fluid (CSF) IgG level, IgG index, 24-h IgG synthesis rate, and positive rate of oligoclonal bands (OCB) were lower in MOGAD patients than in the MS group. The area under ROC curve (AUC) was 0.939 when combining the relative lymphocyte count and IgG index. Compared to the NMOSD group, the MOGAD group had higher levels of serum complement C4 and lower levels of serum IgG. The AUC of serum C4 combined with FT4 was 0.783.

**Conclusion:**

Statistically significant markers were observed in the laboratory data of MOGAD patients compared to MS/NMOSD patients. The relative lymphocyte count combined with IgG index had excellent diagnostic efficacy for MOGAD and MS, while serum C4 combined with FT4 had better diagnostic efficacy for MOGAD and NMOSD.

## 1. Introduction

Myelin Oligodendrocyte Glycoprotein (MOG) antibody-associated disease (MOGAD) has recently been described as an entity (1) that encompasses a spectrum of autoimmune demyelinating disorders through the Central Nervous System (CNS), distinct from multiple sclerosis (MS) and neuromyelitis optica spectrum disorders (NMOSD). MOGAD is characterized by damage to oligodendrocytes and demyelination as its main features, with clinical manifestations closely related to age. Acute disseminated encephalomyelitis (ADEM) is the most common presentation in young children, while optic neuritis (ON) is more common in children the age of 9 and adults (2).

In 2018, Jarius et al. published an international consensus on MOG antibody detection and established diagnostic criteria for MOGAD (3). The diagnostic criteria for MOGAD are based on positive serum MOG-IgG, combined with clinical presentation and imaging. At the same time, other demyelinating diseases such as multiple sclerosis (MS) and neuromyelitis optica spectrum disorders (NMOSD) must also be excluded.

Due to the poor specificity of the clinical manifestations of MOGAD, it can easily be misdiagnosed as MS, NMOSD or other demyelinating diseases (4). Given the significant differences in treatment and outcome, it is crucial to distinguish between MOGAD, NMOSD and MS. So far, there were quite a lot of studies (5–9) comparing MOGAD with MS and NMOSD in clinical features and imaging findings. In this paper, we conducted a retrospective study between MOGAD and MS, MOGAD and NMOSD from a clinical laboratory perspective. The observed predictive laboratory tests may provide additional tools for the early differential diagnosis of MOGAD.

## 2. Materials and methods

### 2.1. Samples

A total of 26 MOGAD patients who visited Beijing Tiantan Hospital, Capital Medical University, Beijing, China, from January 2018 to March 2022 were selected as research objectives in this single-center, retrospective observational study. 26 MS patients and 26 NMOSD patients were randomly selected as controls. The inclusion criteria (8) for MOGAD were as follows: (1) at least one acute clinical CNS demyelinating event (myelitis, ON or encephalopathy); (2) MOG antibody seropositive by a cell-based assay method; (3) supporting MRI features; (4) exclusion of other diagnoses. The MS diagnosis was determined according to the 2017 McDonald criteria (10) and the NMOSD diagnosis was based on the 2015 International Panel on NMOSD Diagnosis (11). This study was approved by the Ethics Committee of Beijing Tiantan Hospital with approval number KY2022-181-02.

### 2.2. Data collection

Demographic information, including gender and age, was recorded. Clinical characteristics and laboratory data were obtained from the electronic medical records. A Mindray (BC-6900) automated blood cell analyzer from China was used for routine blood counts (whole blood WBC, NEU, LY, NEU% LY%, RBC, PLT). Coagulation parameters (PT, TT, APTT, Fbg) were determined by ACL TOP700 LAS, USA. Serum biochemical parameters (TT3, TT4, FT3, FT4) were measured by Beckman DXI800, USA. Serum immunoglobulins (IgG, IgA, IgM, IgE) and complement C3 and C4, as well as CSF albumin and IgG, IgG index, 24 h intrathecal IgG synthesis rate (24 h IgG) were measured using Siemens BN II, Germany. Percent counts of lymphocyte subsets were determined using BD FACS Canto II analyzer, USA. Cytokines (TNF-α, IL-6, IL-8, IL-2R) were measured by Siemens IMMULITE1000. CSF white cell count was tested by Sysmex XN-2000, Japan. Oligoclonal zone electrophoresis detection was performed using a French Sebia HYDRASYS protein electrophoresis instrument. Antibodies to neurological infections in serum and CSF were detected by a Roche e602, Switzerland. The list of infections includes rubella virus (RUB), toxoplasmosis (TOX), herpes simplex virus type1 and type2 (HSV1, HSV2), cytomegalovirus (CMV), and Epstein–Barr virus (EBV). The detection of the IgG index and24 h IgG were used as an indicator of intrathecal IgG synthesis.

### 2.3. Statistical analysis

All statistical analysis was conducted using SPSS (version 20) software (SPSS, Inc., Chicago, IL). All the analyses were corrected for age, sex, status of blood drawing and thyroid disease by regression analysis and multivariate logistic regression analysis was performed. Receiver operating characteristics (ROC) curve analysis was used to calculate the area under the ROC curve (AUC) and evaluate the diagnostic value of meaningful tests for differentiating MOGAD from NMOSD and MS.

## 3. Results

### 3.1. Demographic features

Demographic and clinical characteristics of the study participants are summarized in Table 1. The MOGAD group consisted of 26 patients with a mean onset age of 29.8 years and a female% of 57.7. The MS group included patients with a mean onset age of 33.3 years and a female% of 65.4. The NMOSD group included patients with a mean onset age of 41.8 years and a female% of 73.1, All patients with MOGAD and MS, and 85% NMOSD patients were treated with immunotherapies. Most patients in our study were in relapse at the time of blood withdrawal.

**Table 1:** The detailed findings of demographic, clinical features, and treatment in MOGAD, MS, and NMOSD patients.

	MOGAD	MS	NMOSD
Male/Female, *n*/*n*(%female)	11/15 (57.7)	9/17 (65.4)	7/19 (73.1)
Age at onset, mean (SD)	29.8 (16.1)	33.3 (10.9)	41.8 (16.3)
Disease duration, years, median (IQR)	2.4 (0.4–7.3)	1.2 (0.1–4.9)	1.0 (0.1–4.0)
Status at time of blood draw, *n*			
Onset	4	4	3
Relapse	19	17	21
Remission	3	5	2
Clinical symptom, *n*			
ON	13	8	8
Myelitis	11	26	25
Encephalopathy	24	11	8
Immunotherapy, *n*	Steroids (19)	Steroids (20)	Steroids (18)
	Intravenous immunoglobulins (3)	Intravenous immunoglobulins (4)	Rituximab (3)
	Plasma exchange (2)	Teriflunomide (1)	Tocilizumab (1)
	Mycophenolate mofetil (3)	Siponimod (9)	No information (4)
	Rituximab (12)	Fingolimod (2)	
	Tocilizumab (1)	Rituximab (5)	
Concomitant autoimmune disease, *n*	IgA nephropathy (1)	0	Systemic lupus erythematosus (1)
	Anaphylactic purpura (1)		Sicca syndrome (1)
Thyroid disease, *n*	Thyroid nodules (1)	Thyroid nodules (2)	Thyroid nodules (3)

### 3.2. Comparison of whole blood/plasma/serum and CSF detailed findings among patients with MOGAD and MS

**Figure 1:**
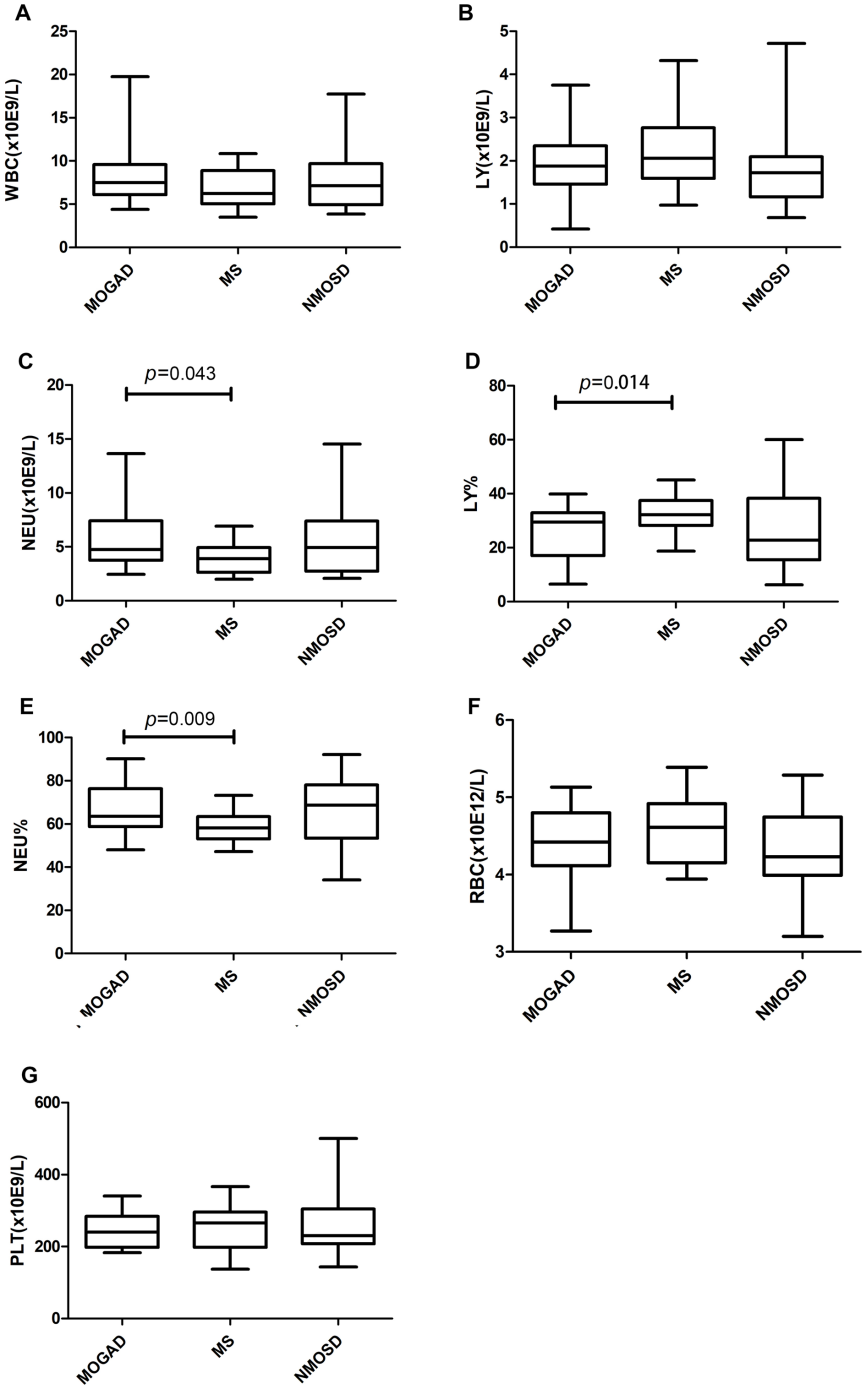
Routine blood counts **(A–G)** in patients with MOGAD, MS and NMOSD. Data are presented as box plots. WBC, white blood cell; LY, lymphocyte; NEU, neutrophile granulocyte; RBC, red blood cell; PLT, platelet.

**Figure 2:**
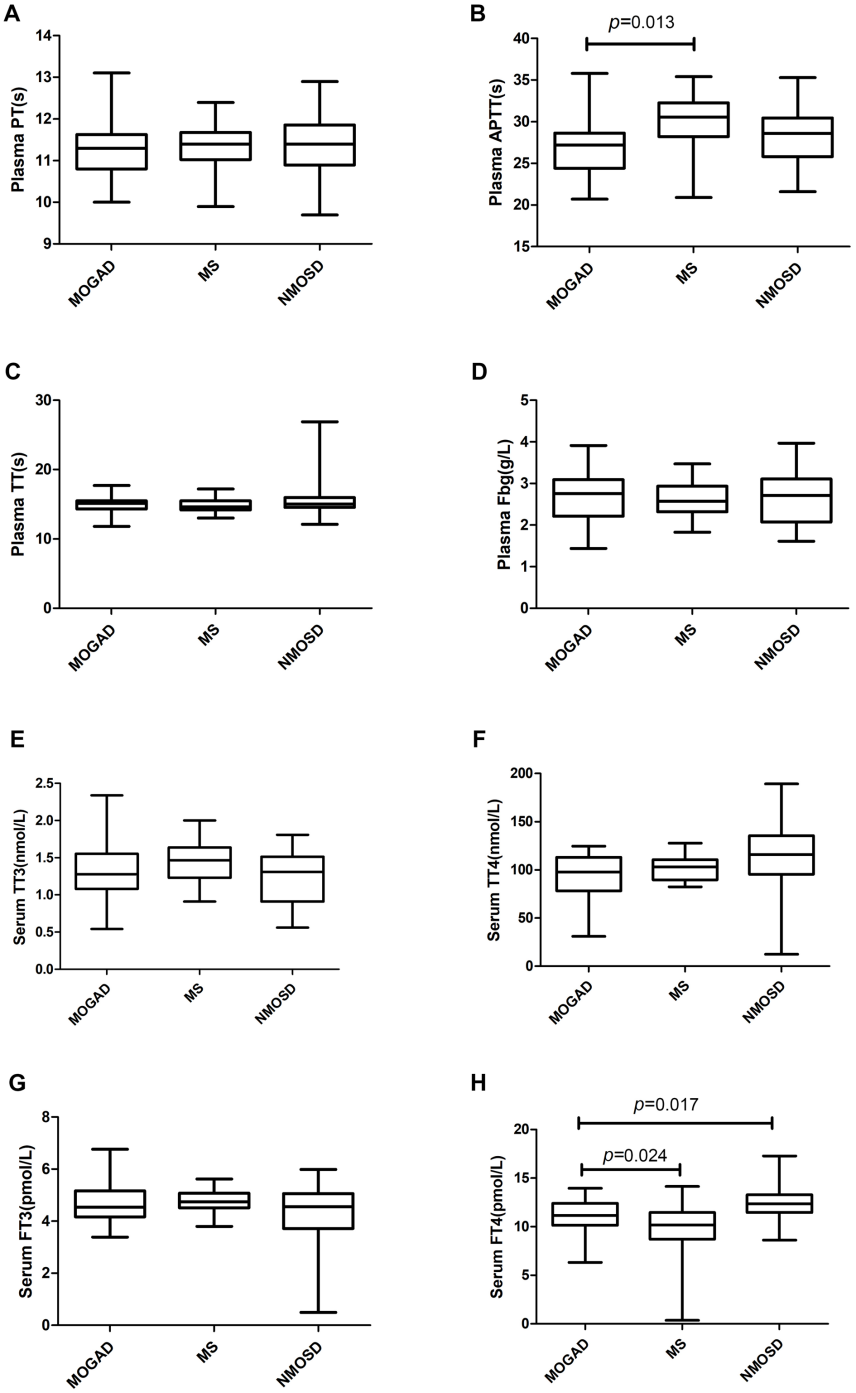
Coagulation parameters **(A–D)** and biochemical parameters **(E–H)** in patients with MOGAD, MS and NMOSD. Data are presented as box plots. PT, prothrombin time; APTT, activated partial thromboplastin time; TT, thrombin time; Fbg, fibrinogen; TT3 and TT4, total triiodothyronitric acid and thyroxine;FT3 and FT4, free triiodothyronitric acid and thyroxine.

**Figure 3:**
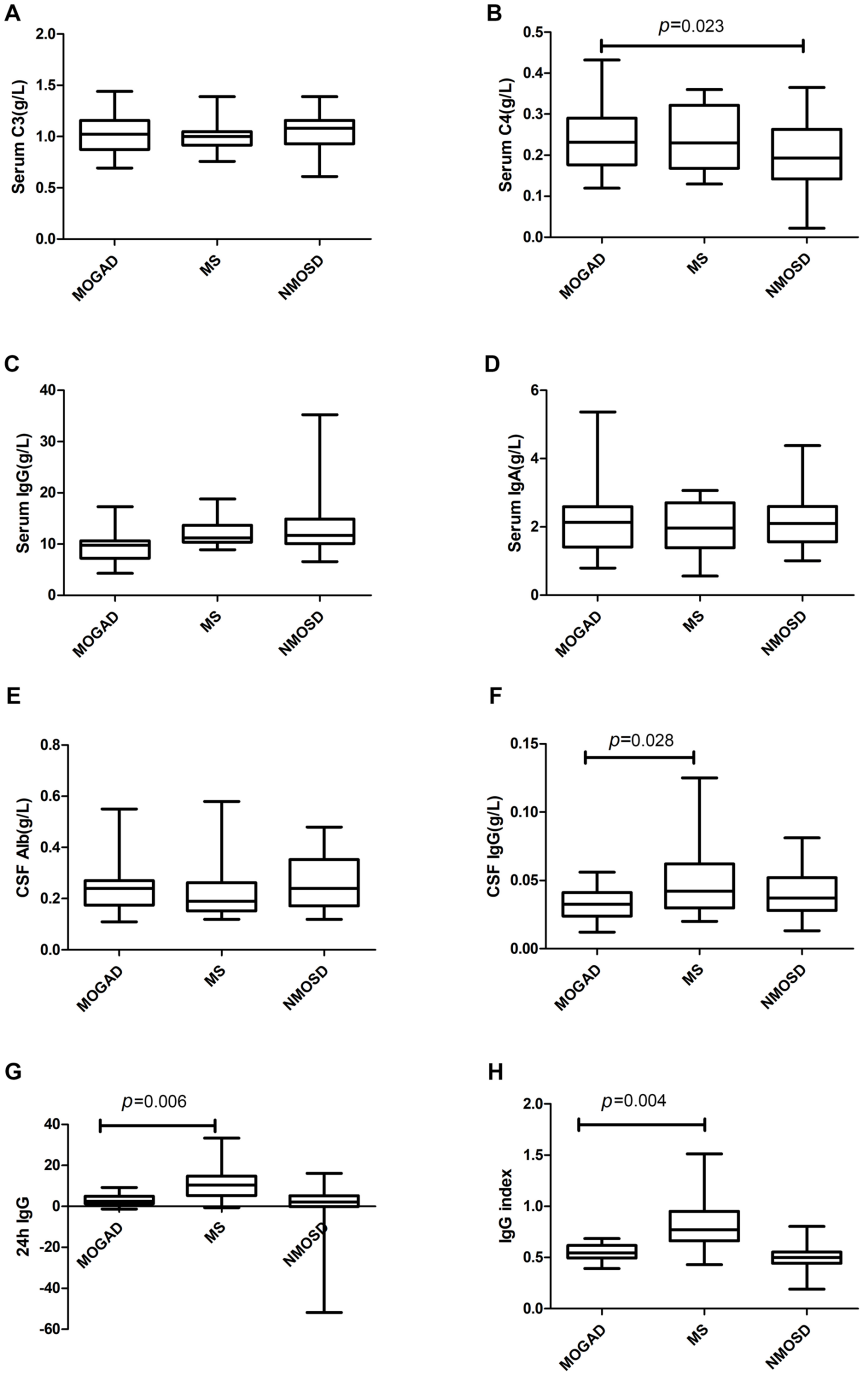
Complement **(A,B)**, immunoglobulin **(C,D)** and CSF parameters **(E–H)** in patients with MOGAD, MS and NMOSD. Data are presented as box plots.

Details of the indicators are given in Supplementary Table 1 and Figures 1–3 present a summary of the notable findings in the serum/plasma and CSF of the study participants. Absolute and relative neutrophil counts and relative lymphocyte counts also showed significant differences between the two groups (Figures 1C–E). Significant differences were observed between the two groups in relation to CSF-IgG, the IgG index and 24 h IgG (Figures 3F–H). The MOGAD group had a lower serum level of FT4 (Figure 2H) and a higher plasma level of APTT (Figure 2B), with a statistically significant difference in comparison to the MS group. The other items that did not show a statistically significant difference are shown in Supplementary Figures 1, 2.

In terms of OCB seropositivity, there was a significant difference between the MOGAD and MS groups (*p* = 0.035). The MOGAD group showed no abnormal IgG index (IgG index > 0.7), whereas the MS group exhibited a higher rate of abnormal IgG index (*p* < 0.001), as displayed in Table 2.

**Table 2:** Intrathecal synthesis index in MOGAD group and control group.

	MOGAD group	MS group	NMOSD group	*P^a^*	*P^b^*
OCB positivity, *n* (%)	5 (26.3)	9 (59.1)	4 (18.2)	0.035	0.530
IgG index > 0.7, *n* (%)	0 (0)	14 (60.9)	2 (10.0)	<0.001	0.129

^a^MOGAD group vs. MS group. ^b^MOGAD group vs. NMOSD group.

The results of ROC analysis for discriminating between MOGAD and MS group were presented in Table 3. A combined ROC curve analysis of relative lymphocyte count and IgG index yielded the highest ROC-AUC of 0.939 (with a sensitivity of 85.0% and specificity of 90.9%). A multivariate model using serum LY% and IgG index was performed and it was found that the IgG index was the most influential variable (OR (95%CI), 0.980 (0.967,0.994), *p* = 0.004) in the differentiation of MOGAD patients from MS patients.

**Table 3:** The discriminating ability of meaningful findings in MOGAD and MS.

	95%CI	AUC	Sensitivity	Specificity	Youden index	*p*	Cut-off value
Whole blood NEU	0.529~0.848	0.689	68.2%	65.0%	0.332	0.037	4.23 × 10E9/L
Whole blood LY%	0.530~0.850	0.690	95.0%	45.5%	0.405	0.035	24.15%
Whole blood NEU%	0.569~0.877	0.723	45.5%	95.0%	0.405	0.014	67.50%
Plasma APTT	0.556~0.885	0.720	60.0%	86.4%	0.464	0.015	29.5 s
Serum FT4	0.575~0.884	0.730	45.5%	95.0%	0.405	0.011	11.82 pmol/L
CSF IgG	0.532~0.854	0.693	45.0%	91.0%	0.359	0.032	0.05 g/L
IgG index	0.772~1.000	0.886	80.0%	91.0%	0.709	<0.001	0.65
24 h IgG	0.705~0.963	0.834	80.0%	77.3%	0.573	0.000	4.82
LY% combined IgG index	0.869~1.000	0.939	85.0%	90.9%	0.759	<0.001	/

### 3.3. Comparison of whole blood/plasma/serum and CSF detailed findings among patients with MOGAD and NMOSD

The serum levels of FT4 (Figure 2H) were significantly lower in the MOGAD group than in the NMOSD group. In contrast, the serum level of C4 was higher in the MOGAD group (refer to Figure 3B). OCB positivity was 26.3% in the MOGAD group and 18.2% in the NMOSD group, with no significant difference found between the two groups (*p* = 0.530).

Table 4 presents the results of ROC analysis for differentiating between the MOGAD and NMOSD groups. A combined ROC curve analysis of serum C4 and TT4, yielded a maximum ROC-AUC of 0.783 with a sensitivity of 54.2% and specificity of 95.8%. We built a multivariate model using serum C4 and FT4 and found that C4 was the most important variable (OR (95%CI), 4.037 (1.326,12.284), *p* = 0.014) in differentiating MOGAD patients from NMOSD patients.

**Table 4:** The discriminating ability of meaningful findings in MOGAD and NMOSD.

	95%CI	AUC	Sensitivity	Specificity	Youden index	*p*	Cut-off value
Serum C4	0.503~0.808	0.655	69.2%	66.7%	0.359	0.06	0.532 g/L
Serum FT4	0.532~0.830	0.681	61.5%	70.8%	0.324	0.028	12.02 pmol/L
C4 combined FT4	0.668~0.919	0.783	54.2%	95.8%	0.500	0.001	/

### 3.4. Positivity rate of viral infections for MOGAD, MS, and NMOSD

Table 5 shows the positivity rate for viral infections in MOGAD, MS and NMOSD. The highest prevalence of RUB, CMV and EBV was found in the sera of MOGAD and MS patients, followed by HSV1, HSV2 and TOX. HSV was detected in the serum of about 50% of NMOSD patients. Viral antibodies were rarely detected in CSF. Only CMV (42.9%) was found in the CSF of patients with MOGAD. CMV was the most frequently detected virus in the CSF of MS (47.6%) and NMOSD (66.7%) patients.

**Table 5:** The positivity rate (%) of viral infections.

	RUBIgG	TOXIgG	CMVIgG	HSV-1	HSV-2	EBVCA^c^-IgG	EBNA^d^-IgG
MOGAD_Serum	90.5	9.5	81.0	66.7	19.0	85.7	81.0
MOGAD_CSF	0.0	0.0	42.9	0.0	0.0	0.0	0.0
MS_Serum	66.7	9.5	95.2	76.2	9.5	100.0	90.5
MS_CSF	4.8	0.0	47.6	0.0	0.0	0.0	4.8
NMOSD_Serum	26.7	13.3	33.3	46.7	6.7	33.3	33.3
NMOSD_CSF	20.0	0.0	66.7	6.7	0.0	13.3	13.3

^c^EBV viral capsid antigen. ^d^EBV viral nuclear antigen.

## 4. Discussion

MOGAD accounts for 1.2%–6.5% of all demyelinating diseases in adult patients and 40% in children (12). The pathogenesis of this disease is multifactorial and complex, and it has a diverse clinical presentation. MOGAD can be monophasic or relapsing, with major symptoms including ON, myelitis, or encephalopathy. MOG-IgG is one of the diagnostic indicators of MOGAD. In terms of clinical application, however, it has some limitations. First, the specificity of MOG-IgG for MOGAD needs to be explored. For example, when tested in patients with typical clinical and radiological features of multiple sclerosis, there is a certain degree of false-positive rate (13). Therefore, MOG-IgG testing in appropriate populations is preferred. Second, live cell-based MOG-IgG assays have the highest specificity. However, some commercial assays use fixed cells. Last, MOG-IgG assay results are reported in an inconsistent format with qualitative or quantitative expressions (14), which poses a problem for the precise interpretation of clinical results. In this study, we focused on laboratory data in search of relevant indicators for differential diagnosis of MOGAD.

A total of 26 patients with MOGAD were selected for this study with a slight female preponderance (male-to-female ratio of 1:1.36). The UK cohort (15) also showed a slight female preponderance of 62%, with an average age of onset of 31 years (range 3–69 years). In a multi-center study (16) involving at least three US race/ethnicities (white, black, Asian and other), the proportion of women was as high as 85% (21/25). The prevalence of MOGAD varies between men and women in different studies. MOGAD is a rare disease with low incidence, and none of the above studies had a sufficiently large population to comprehensively reflect the gender and age distribution.

Increasing evidence suggests that some MOGAD patients have initial phenotypes suggestive of MS (17) and they could have typical MS attacks at onset. In this study, ROC curve analysis demonstrated that combined use of LY% and IgG index yielded the highest diagnostic efficacy (AUC of 0.939) in differentiating between MOGAD and MS.

We conducted a differential analysis of relevant laboratory data between the two groups and discovered that patients in the MOGAD group differed from the MS group in terms of blood counts. MS, the most common inflammatory demyelinating disease of CNS, is characterized by lymphocyte infiltration and inflammation of the CNS white matter (18). B lymphocytes are capable of migrating across the blood–brain barrier to produce oligoclonal bands, while T lymphocytes can be activated to produce inflammatory cytokines (19).

The IgG index is used to measure intrathecal antibody production. Increased IgG index or CSF-restricted OCB is a characteristic feature of MS, observed in over ∼85% of cases (6). In the present study, CSF IgG levels, IgG index, 24 h IgG, and OCB positivity were lower in MOGAD group than in the MS group. The rate of OCB positivity in the MOGAD group in this study was 26.3% (5/19), which is higher than in the Caucasian descent (where approximately 13% of MOGAD patients were OCB positive) (13). Studies in the UK, Germany and Italy have reported MOGAD positivity rates of around 5%–20%. A multicenter study in the United States (14) showed an OCB positivity rate of 20% (5/25) in the MOGAD group, which is slightly lower than the results of this study. Variation in OCB positivity in the MOGAD group between studies may be related to race (13, 20, 21).

MOG-IgG is a supporting biological marker for the diagnosis of MOGAD, but approximately 40% of NMOSD patients who are negative for aquaporin-4(AQP4)-IgG will be positive for MOG-IgG (22). There appear to be close similarities in disease presentation between MOGAD and NMOSD. Among patients with MOGAD, 23% of adults and 31% of children met the seronegative criteria for NMOSD (23). In our retrospective study, combination of serum FT4 levels with complement component C4 demonstrated acceptable diagnostic efficacy for both MOGAD and NMOSD (with an AUC of 0.783).

We found serum FT4 were significantly lower in the MOGAD group than in the NMOSD group and there were no significant differences in terms of routine blood tests, lymphocyte subsets, cytokines, immunoglobulins, and 24hIgG. Thyroid hormones are important hormones that maintain the growth and development of the human nervous system. Related EAE (experimental autoimmune encephalomyelitis) animal experiments (24) showed that thyroid hormones and thyroid-mimetic drugs can induce oligodendrocyte differentiation and promote myelin regeneration in mice. The glycoprotein MOG is specifically expressed on the myelin surface of oligodendrocytes, and the relatively low level of thyroid hormone in the MOGAD group may be related to the demyelination of oligodendrocytes.

A study by Lin et al. (25) demonstrated that the plasma concentrations of C3 and C4 were higher in the MOGAD group (*n* = 22) than in the NMOSD group (*n* = 73), and the area under the ROC curve for C3 and C4 was 0.731 and 0.645, respectively. Additionally, our study found that C4 was higher in the MOGAD group than in the NMOSD group, with an area under the ROC curve of 0.655 (Table 5), consistent with the findings of Liuyu Lin et al. Similarly, higher serum C4 concentrations were found in MOGAD patients (*n* = 15) than in NMOSD patients (*n* = 16) in the research by Florence Pache et al. (26). Another study by Christian W. Keller et al. compared the MOGAD group (*n* = 109) with the MS group (*n* = 34), NMOSD group (*n* = 13), and healthy controls (*n* = 16), indicating that complement indicators C3a, C5a, SC5b9, Ba, and Bb had the highest concentrations in MOGAD, suggesting that the activated complement system plays a significant role in the pathogenesis of MOGAD (27). C3 and C4 activation is a key factor in activating the entire complement system (28). Several studies have reported the presence of elevated complement in MOGAD patients, but it is unclear whether complement is involved in the key pathogenic link of MOGAD (29, 30), necessitating further studies.

Virus infections have been proposed as potential triggers of MOGAD and MS. EBV infection is known to be associated with the onset of MS. In recent years, there have also been cases of MOGAD reporting common viruses as EBV infection (31), as well as rare virus such as Jamestown Canyon virus (32). In our study, MOGAD patients were more likely to be infected with RUB, CMV and EBV. The serum EBV positivity for MOGAD was 85.7% in the present study, which was higher than another study (16) on children with MOGAD patients (11/25, 44%). To our knowledge, there have been few studies reporting on MOGAD patients infected with RUB and CMV. In our study, only CMV was detected in the CSF of MOGAD, much lower than the virus species detected in serum. Currently, the pathogenesis of MOGAD has not been elucidated. As the MOG antigen is exclusively expressed in the CNS, the mechanism involved in the production of MOG-IgG is also unclear. It has been suggested (12) that the blood–brain barrier is compromised during exposure to exogenous infections, causing MOG antigens to leak into the peripheral blood and generate substantial amounts of MOG-IgG. Concurrently, MOG-specific B cells enter the CNS and produce corresponding antibodies. However, some researchers dispute this notion. According to scientists from Sendai University, Japan (33), MOG-IgG antibodies in CSF are more often produced via intrathecal synthesis, distinct from the source of AQP4-IgG antibodies. This intrathecal synthesis does not always correspond to IgG index and OCB, but is indicated by Q_MOG-IgG_ (the ratio of CSF MOG-IgG titer to serum MOG-IgG titer). Unfortunately, the data limitations of this study preclude the calculation of Q_MOG-IgG_, so the issue of MOG-IgG production and origin remains unresolved.

In summary, this study examined MOGAD, MS, and NMOSD using laboratory assessments and discovered several statistically significant differences in tests. Analysis of diagnostic efficacy demonstrated that combining whole blood LY% with IgG index showed better diagnostic value for MOGAD and MS, while serum C4 in combination with FT4 had superior diagnostic efficacy for MOGAD and NMOSD. MOGAD shares clinical and imaging manifestations with various inflammatory demyelinating diseases, and the findings of this study could provide a laboratory foundation for distinguishing MOGAD from MS and NMOSD.

Our study has some limitations. First, the number of patients in this study was small, and the conclusions will need further large-scale studies. In addition, the data collected may have been influenced by a few factors, including the immunotherapy and the disease status at the time of sampling.

## Data availability statement

The original contributions presented in the study are included in the article/Supplementary material, further inquiries can be directed to the corresponding author.

## Ethics statement

The studies involving humans were approved by the Ethics Committee of Beijing Tiantan Hospital. The studies were conducted in accordance with the local legislation and institutional requirements. This study is a retrospective analysis using information from electronic medical records. Our study does not take samples directly from patients. Written informed consent for participation was not required from the participants or the participants’ legal guardians/next of kin in accordance with the national legislation and institutional requirements.

## Author contributions

All authors listed have made a substantial, direct, and intellectual contribution to the work and approved it for publication.

## Funding

This work was supported by the National Science Foundation of China (No. 82002198), Beijing Hospitals Authority Youth Programme (code: QML20210507), Beijing Hospitals Authority’s Ascent Plan (DFL20220505), and Beijing High-level Public health technical Personnel Training program (2022-2-013).

## Conflict of interest

The authors declare that the research was conducted in the absence of any commercial or financial relationships that could be construed as a potential conflict of interest.

## Publisher’s note

All claims expressed in this article are solely those of the authors and do not necessarily represent those of their affiliated organizations, or those of the publisher, the editors and the reviewers. Any product that may be evaluated in this article, or claim that may be made by its manufacturer, is not guaranteed or endorsed by the publisher.

## Supplementary material

The Supplementary material for this article can be found online at: https://www.frontiersin.org/articles/10.3389/fneur.2023.1187824/full#supplementary-material
