# Supplementary figures and images for: A retrospective study of myelin oligodendrocyte glycoprotein antibody-associated disease from a clinical laboratory perspective
Source: Front Neurol. 2023 Sep 12;14:1187824. doi: 10.3389/fneur.2023.1187824 (PMC10523388; doi:10.3389/fneur.2023.1187824)

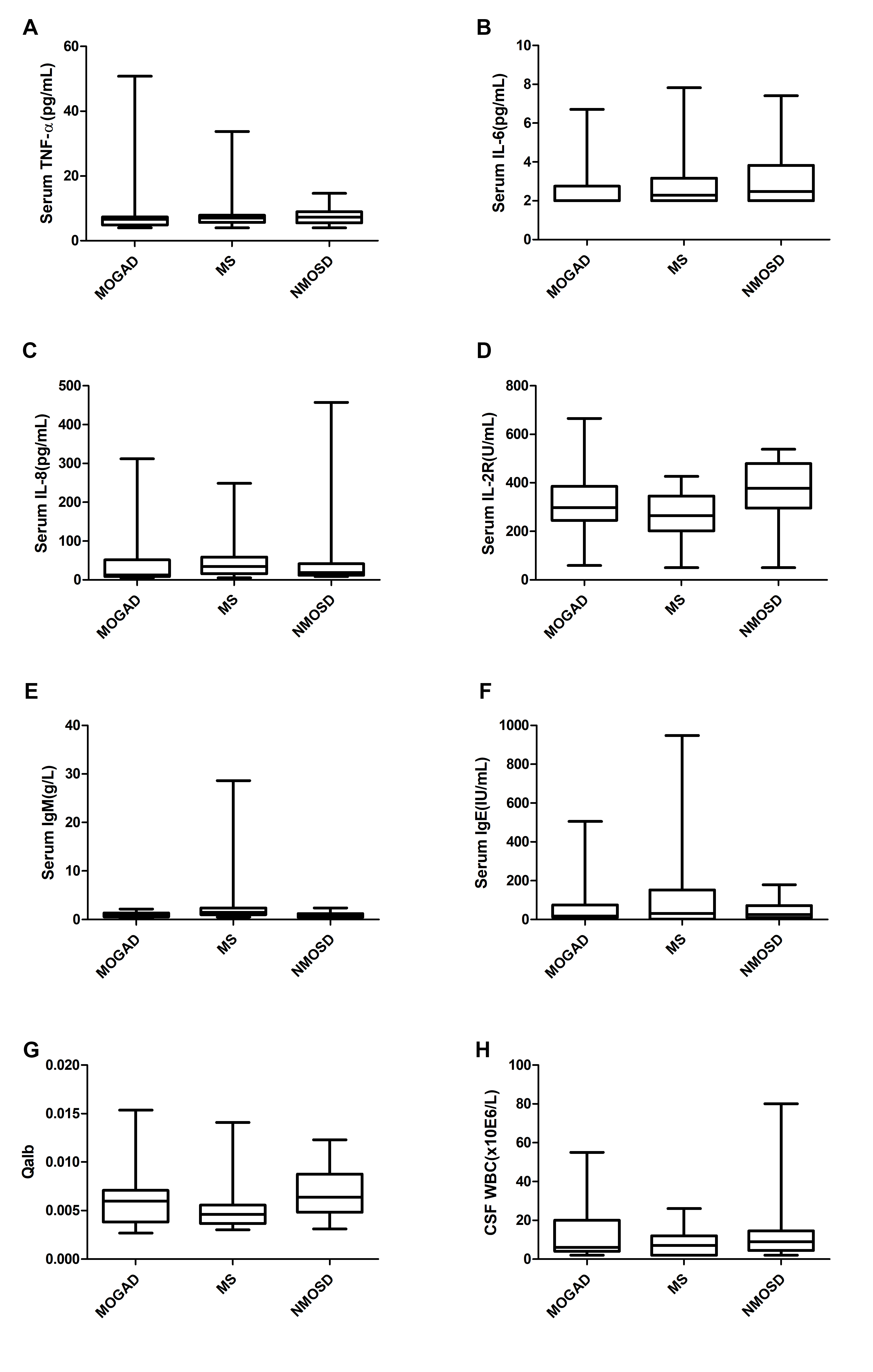

Supplement: Supplementary file 1 [file Image_1.JPEG]

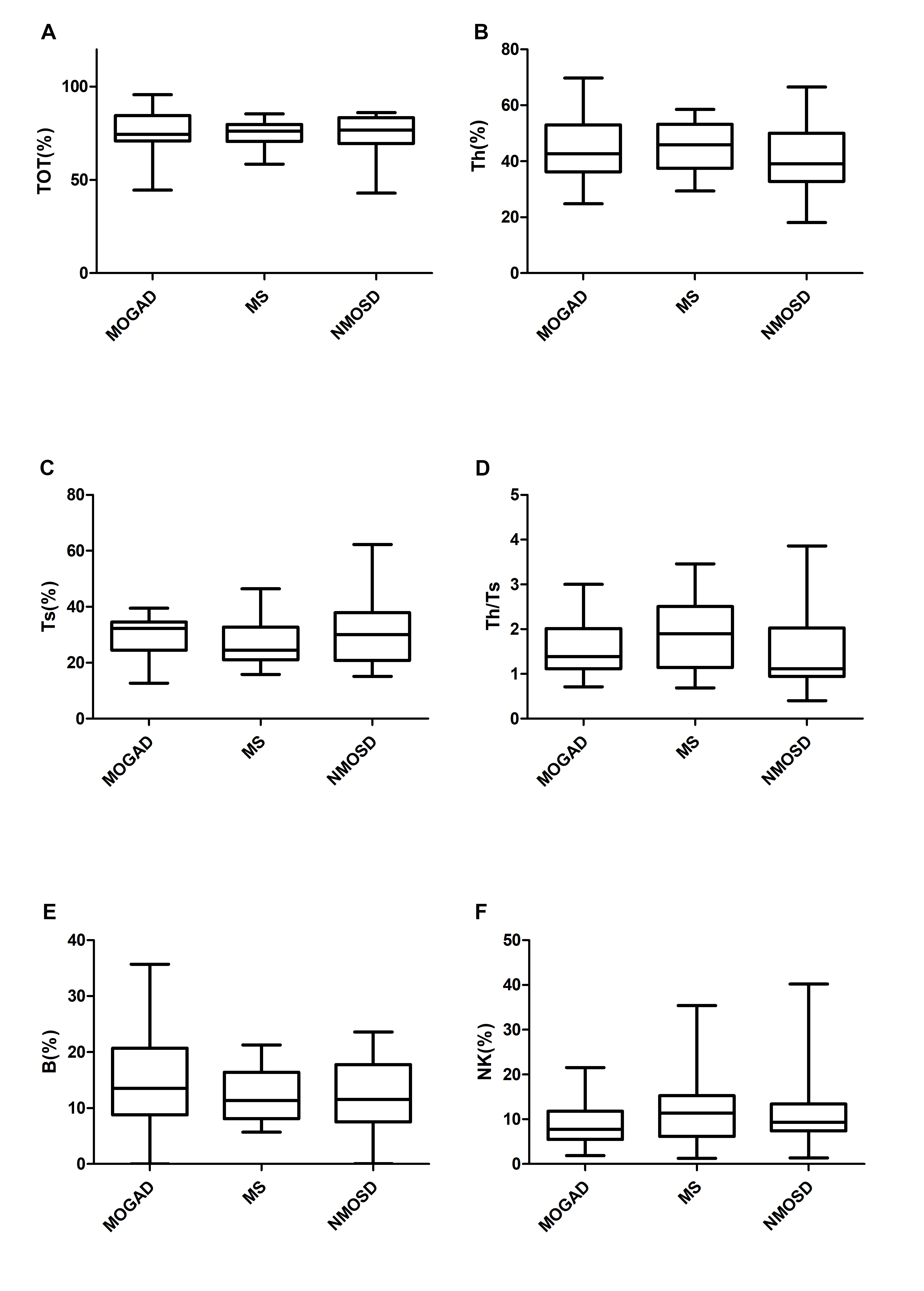

Supplement: Supplementary file 2 [file Image_2.JPEG]
