# Supplementary material for: A retrospective study of myelin oligodendrocyte glycoprotein antibody-associated disease from a clinical laboratory perspective
Source: Front Neurol. 2023 Sep 12;14:1187824. doi: 10.3389/fneur.2023.1187824 (PMC10523388; doi:10.3389/fneur.2023.1187824)
Supplement: Supplementary file 3 [file Table_1.docx]

| Supplementry Table 1. Whole blood/Plasma/serum and CSF indicators among patients with MOGAD, MS and NMOSD and comparisons between disease groups. | | | | | |
| --- | --- | --- | --- | --- | --- |
|  | MOGAD group | MS group | NMOSD group | *P^a^* | *P^b^* |
|  | *M（P25，P75）* | *M（P25，P75）* | *M（P25，P75）* |  |  |
| **Whole blood** |  |  |  |  |  |
| WBC (x10E9/L) | 7.49（6.09,9.59） | 6.23（5.05,8.89） | 7.13（4.93,9.69） | 0.294 | 0.992 |
| LY (x10E9/L) | 1.88（1.46,2.34） | 2.06（1.60,2.77） | 1.72（1.16,2.10） | 0.548 | 0.993 |
| NEU(x10E9/L) | 4.74（3.75,7.40） | 3.89（2.64,4.92） | 4.93（2.74,7.39） | **0.043** | 0.604 |
| LY% | 29.45（17.03,32.90） | 32.15（28.23,37.50） | 22.80（15.40,38.30） | **0.014** | 0.793 |
| NEU% | 63.55（58.83,76.25） | 58.15（53.00,63.4） | 68.70（53.40,78.05） | **0.009** | 0.845 |
| RBC(x10E12/L) | 4.42（4.12,4.80） | 4.61（4.16,4.92） | 4.23（3.99,4.75） | 0.104 | 0.439 |
| PLT(x10E12/L) | 240.00（197.50,284.25） | 265.50（197.75,295.50） | 304.00（230.00,325.80） | 0.938 | 0.431 |
| ToT% | 74.47（70.93,84.43） | 76.12（70.69,79.61） | 76.77（69.57,83.35） | 0.721 | 0.809 |
| Th% | 42.67（36.22,52.97） | 45.93（37.49,53.26） | 39.09（32.75,50.03） | 0.781 | 0.424 |
| Ts% | 32.25（24.52,34.59） | 24.53（21.03,32.68） | 30.02（20.82,37.92） | 0.323 | 0.338 |
| Th/Ts | 1.39（1.12,2.01） | 1.90（1.15,2.51） | 1.12（0.95,2.03） | 0.196 | 0.863 |
| B% | 13.55（8.80,20.73） | 11.34（8.09,16.40） | 11.51（7.52,17.77） | 0.415 | 0.214 |
| NK% | 7.73（5.52,11.79） | 11.36（6.21,15.26） | 9.30（7.39,13.38） | 0.187 | 0.137 |
| **Plasma** |  |  |  |  |  |
| PT (s) | 11.30（10.80,11.63） | 11.40（11.03,11.68） | 11.40（10.90,11.85） | 0.598 | 0.294 |
| APTT (s） | 27.20（24.4,28.63） | 30.55（28.23,32.25） | 28.60（25.80,30.45） | **0.013** | 0.096 |
| TT (s) | 15.10（14.33,15.53） | 14.60（14.20,15.48） | 15.00（14.55,15.59） | 0.523 | 0.66 |
| Fbg (g/L) | 2.76（2.22,3.09） | 2.58（2.32,2.94） | 2.71（2.08,3.11） | 0.853 | 0.853 |
| **Serum** |  |  |  |  |  |
| TT3 (nmol/L) | 1.28（1.08,1.56） | 1.47（1.23,1.47） | 1.31（0.91,1.51） | 0.243 | 0.86 |
| TT4 (nmol/L) | 97.91（78.33,113.09） | 103.12（89.66,110.80） | 116.03(95.41,135.62) | 0.168 | 0.148 |
| FT3 (pmol/L) | 4.55（4.17,5.16） | 4.75（4.51,5.08） | 4.56（3.72,5.06） | 0.256 | 0.733 |
| FT4 (pmol/L） | 11.16（10.15,12.38） | 10.17（8.72,11.47） | 12.34(11.46,13.29) | **0.024** | **0.017** |
| C3 (g/L) | 1.02（0.87,1.16） | 1.08（0.93,1.16） | 1.00（0.92,1.05） | 0.291 | 0.291 |
| C4 (g/L) | 0.23（0.18,0.29） | 0.23（0.17,0.32） | 0.19（0.14,0.26） | 0.124 | **0.023** |
| IgG (g/L) | 11.10（8.19,12.40） | 10.85（9.91,13.13） | 11.35（9.96,14.25） | 0.087 | 0.204 |
| IgA (g/L) | 2.14（1.42,2.59） | 1.97（1.40,2.70） | 2.10（1.57,2.60） | 0.386 | 0.906 |
| IgM (g/L) | 0.86（0.54,1.36） | 1.53（0.98,4.02） | 0.75（0.50,1.20） | 0.198 | 0.448 |
| IgE (IU/mL) | 18.25（10.35,75.83） | 30.90（2.90,152.75） | 26.25（10.53,71.78） | 0.176 | 0.863 |
| TNFα(pg/mL) | 6.63（4.86,7.38） | 7.00（5.70,7.88） | 7.27（5.58,8.95） | 0.904 | 0.976 |
| IL-6 (pg/mL) | 2.00（2.00,2.75） | 2.28（2.00,3.16） | 2.48（2.00,3.83） | 0.233 | 0.075 |
| IL-8 (pg/mL) | 12.75（8.54,52.08） | 34.2（15.80,59.10） | 18.40（12.08,41.25） | 0.631 | 0.797 |
| IL-2R (U/mL) | 297.60（245.25,385.5） | 265.00（202.00,345.00） | 377.50（296.50,478.75） | 0.138 | 0.171 |
| **CSF** |  |  |  |  |  |
| WBC (x10E6/L) | 6.00（4.00,20.00） | 7.00（2.00,12.00） | 9.00（5.00,19.00） | 0.181 | 0.605 |
| Qalb | 0.006（0.004,0.007） | 0.005（0.004,0.006） | 0.006（0.005,0.008） | 0.320 | 0.566 |
| CSF Alb (g/L) | 0.24（0.18,0.27） | 0.19（0.15,0.26） | 0.24（0.17,0.35） | 0.746 | 0.658 |
| CSF IgG(g/L) | 0.03（0.02,0.04） | 0.04（0.03,0.06） | 0.04（0.03,0.05） | **0.028** | 0.221 |
| IgG index | 0.54（0.49,0.62） | 0.77（0.66,0.95） | 0.50（0.44,0.55） | **0.006** | 0.252 |
| 24h IgG | 2.49（1.18,4.85） | 10.37（5.19,14.75） | 2.17（-0.11,5.16） | **0.004** | 0.561 |

^a^MOGAD group Versus MS group. ^b^MOGAD group Versus NMOSD group. *P*<0.05 shown in bold.
